# Long-term outcomes following a pathological complete response at the primary tumor site after preoperative therapy in metastatic colorectal cancer

**DOI:** 10.1093/oncolo/oyag025

**Published:** 2026-02-03

**Authors:** Dakui Luo, Yufei Yang, Yikuan Chen, Qingguo Li, Chunkang Yang, Xinxiang Li

**Affiliations:** Department of Colorectal Surgery, Fudan University Shanghai Cancer Center, Shanghai 200032, China; Department of Oncology, Shanghai Medical College, Fudan University, Shanghai 200032, China; Department of Colorectal Surgery, Fudan University Shanghai Cancer Center, Shanghai 200032, China; Department of Oncology, Shanghai Medical College, Fudan University, Shanghai 200032, China; Department of Colorectal Surgery, Fudan University Shanghai Cancer Center, Shanghai 200032, China; Department of Oncology, Shanghai Medical College, Fudan University, Shanghai 200032, China; Department of Colorectal Surgery, Fudan University Shanghai Cancer Center, Shanghai 200032, China; Department of Oncology, Shanghai Medical College, Fudan University, Shanghai 200032, China; Department of Gastrointestinal Surgical Oncology, Clinical Oncology School of Fujian Medical University, Fujian Cancer Hospital, Fuzhou 350014, China; Department of Colorectal Surgery, Fudan University Shanghai Cancer Center, Shanghai 200032, China; Department of Oncology, Shanghai Medical College, Fudan University, Shanghai 200032, China

**Keywords:** pathological complete response, metastatic colorectal cancer, preoperative therapy

## Abstract

**Background:**

A pathological complete response (pCR) following neoadjuvant chemoradiation (CRT) is associated with a favorable prognosis in patients with locally advanced rectal cancer. Patients with metastatic colorectal cancer (mCRC) often receive systemic therapy, and some patients undergo primary tumor resection after preoperative therapy. However, whether oncological outcomes remain favorable in patients with mCRC and pCR in the primary tumor site (ypT0N0M1) following preoperative therapy is unknown.

**Patients and methods:**

Patients with mCRC who underwent preoperative therapy followed by primary tumor resection between March 2014 and August 2023, and in whom pCR was confirmed at the primary tumor site, were retrospectively included in this study. The outcome variables investigated were patient demographics, overall survival (OS), and progression-free survival (PFS).

**Results:**

We included 57 patients who met the inclusion criteria. The median follow-up was 27.1 (range, 7.9-120.6) months. The 2-year PFS and 3-year OS rates in all the patients were 62.2% and 84.5%, respectively. Patients with a primary tumor site in the colon (*n* = 29) had superior OS (*P* = 0.039) and a trend toward superior PFS (*P* = .149) compared to those with a primary site in the rectum (*n* = 28).

**Conclusion:**

Patients with mCRC following preoperative therapy, particularly those with colon cancer, who experienced a pCR in the primary tumor site have a favorable prognosis.

Implications for Practice This study highlights the favorable oncological prognosis of patients with metastatic colorectal cancer (mCRC) with a pathological complete response (pCR) at the primary tumor site following neoadjuvant therapy, providing critical clinical context for managing this select subgroup. Notably, the data demonstrate that colon cancer patients with primary tumor pCR exhibit superior survival outcomes compared to their counterparts with rectal cancer. These findings advocate for tumor location-tailored therapeutic strategies when managing patients with mCRC who attain primary tumor pCR, as treatment responses and long-term prognosis may differ substantially based on whether the primary lesion arises in the colon or rectum. By integrating tumor location into clinical decision-making, clinicians can refine therapeutic strategies, balancing efficacy, toxicity, and quality of life, to maximize survival benefits for this distinct patient subgroup.

## Introduction

Colorectal cancer (CRC) is a leading cause of cancer morbidity and mortality worldwide. Among these cancer types, locally advanced rectal cancer (LARC) represents a major clinical challenge. The standard therapy for LARC is neoadjuvant chemoradiotherapy followed by total mesorectal excision. Recently, neoadjuvant short-course radiotherapy followed by immunochemotherapy demonstrated improved efficacy in patients with proficient mismatch repair or microsatellite stability (pMMR/MSS).^1^^,^^2^ Neoadjuvant therapy improves local tumor control and sphincter preservation in most cases. Some patients with pathological complete response (pCR) have evident improvement in long-term outcomes.^3^^,^^4^ Neoadjuvant chemotherapy is commonly used in locally advanced colon cancer (LACC), particularly in high-risk relapsed LACC. Current evidence indicates that although neoadjuvant chemotherapy does not improve the prognosis of colon cancer, it offers certain advantages in pathological downstaging. Additionally, a small subset of patients can achieve a pCR. For colon cancer specifically, the pCR rate following neoadjuvant therapy is reported to range from 3% to 7% in the literatures.^5^^,^^6^ On the other hand, approximately 15%-20% of patients with CRC are diagnosed with synchronous distant metastasis,^7^ which usually has a poor prognosis. In addition, metastatic CRC (mCRC) exhibits significant heterogeneity.^8^ Accordingly, the therapeutic options for mCRC have changed significantly in recent years.^9^ In general, systemic therapy remains the primary therapeutic option for most patients with mCRC. Some patients in whom systemic therapy demonstrated good efficacy undergo primary tumor resection with or without surgery or local ablative therapy for metastatic lesions. Intriguingly, a small portion of patients can still experience a pCR of the primary site. However, whether oncological outcomes remain favorable in patients with mCRC and pCR in the primary tumor site following preoperative therapy is unknown. This study aimed to investigate the oncological outcomes of ypT0N0M1 CRC with pMMR/MSS. Patients with deficient DNA mismatch repair/microsatellite instability-high (dMMR/MSI-H) mCRC were excluded from our study given higher rates of major and complete response to neoadjuvant therapy, to ensure our results would be specific to the more common population with pMMR/MSS mCRC.^10^

## Materials and methods

### Study population

This was a retrospective observational cohort study of consecutive patients with mCRC who received preoperative therapy followed by resection of the primary lesion and experienced a pCR of the primary tumor in Fudan University Shanghai Cancer Center (FUSCC) (Shanghai, China) between March 2014 and August 2023. Inclusion criteria: (1) histologically confirmed CRC, (2) stage IV disease at diagnosis, confirmed by MRI or CT/PET-CT, (3) preoperative therapy (systemic therapy ± radiotherapy) followed by surgery of the primary site, and (4) postoperative pathology confirmed as pCR of the primary site (ypT0N0). Exclusion criteria: (1) patients with dMMR/MSI-H, (2) incomplete clinical/survival data. A total of 565 patients who received preoperative therapy and surgery for CRC and experienced a pCR were screened. From this group, 507 patients were categorized as ypT0N0M0, and the remaining 58 patients were ypT0N0M1. Among the 58 ypT0N0M1 patients, 1 patient was identified as dMMR and was excluded. As a result, 57 patients were included in the final analysis (Supplementary Figure S1—See online supplementary material for a color version of this figure). The following data were extracted from the FUSCC database: age at diagnosis, sex, g primary tumor location, pretreatment CEA level, preoperative therapy regimen, time interval between the start of preoperative therapy and primary tumor resection, lymph node harvest, status of KRAS, NRAS and BRAF, surgery of metastatic site, metastasis site, and survival data. Elevated CEA is defined as >5.2 ng/mL, as per standard clinical practice. Patients were followed up through outpatient clinic visits and telephone calls. Patients were followed every 3 months for the first 2 years, then every 6 months thereafter, with imaging (CT/MRI) and tumor marker testing to assess disease status. Progression-free survival (PFS) was defined as the time from the initiation of preoperative therapy to the first documented disease progression or cancer-specific death. Overall survival (OS) was defined as the time from the initiation of preoperative therapy to all-cause death.

The study protocol was conducted in accordance with the Declaration of Helsinki and was approved by the ethics committee of FUSCC. Informed consent for the use of clinical and tissue data was obtained from all patients or their legal guardians.

### Treatment

As the study encompassed a long period of time, significant differences were noted in the preoperative treatment regimens, which primarily included traditional chemotherapy, radiotherapy, molecular targeted therapy, and immunotherapy. Variations were also observed in the management of metastatic lesions: some cases involved simultaneous resection of both the primary and metastatic lesions, while others were treated with staged resection. In addition, some metastatic lesions were treated with ablation or stereotactic body radiation therapy, while some had received only systemic treatment because these patients were not amenable to surgery or local therapy.

### Statistical analyses

Survival rates were calculated using the Kaplan–Meier method, and comparisons between the two groups (rectal vs. colon) were performed using the log-rank test. Cox regression was used for univariate and multivariate analyses with HRs and 95% CI. Statistical significance was defined as *P* < .05. All statistical analyses were performed using SPSS version 24.0 (SPSS, Chicago, IL, USA).

## Results

The clinicopathological features of 57 patients with mCRC and pCR of the primary tumor site are shown in Table 1. The most common isolated metastatic site was the liver (*n* = 33, 57.9%), followed by the lung (*n* = 5, 8.8%); 10 patients had multiple-site metastases.

**Table 1.** Patient and tumor characteristics.

Variable	Total (*N* = 57)
**Age (mean ± SD)**	56.7 ± 14.1
**Sex**	
**Male**	35 (61.4%)
**Female**	22 (38.6%)
**Pretreatment CEA**	
**Normal**	29 (50.9%)
**Elevated**	28 (49.1%)
**Location**	
**Rectal**	28 (49.1%)
**Colon**	29 (50.9%)
**Surgical interval (days)**	
**<180**	29 (50.9%)
**≥180**	28 (49.1%)
**Lymph node harvest**	12.2 ± 7.8
**KNB** ^a^ ** status**	
**Wild type**	14 (24.6%)
**Mutation**	21 (36.8%)
**Unknown**	22 (38.6%)
**Radiotherapy**	
**No**	39 (68.4%)
**Yes**	18 (31.6%)
**Metastectomy**	
**No**	26 (45.6%)
**Yes**	31 (54.4%)
**Target therapy**	
**No**	14 (24.6%)
**Yes**	43 (75.4%)
**Site of metastasis**	
**Liver**	33 (57.9%)
**Lung**	5 (8.8%)
**Peritoneal dissemination**	2 (3.5%)
**Extra-regional lymph node**	4 (7.0%)
**Bone**	3 (5.3%)
**Multiple sites**	10 (17.5%)

a KNB: KRAS, NRAS, BRAF.

**Figure 1.**
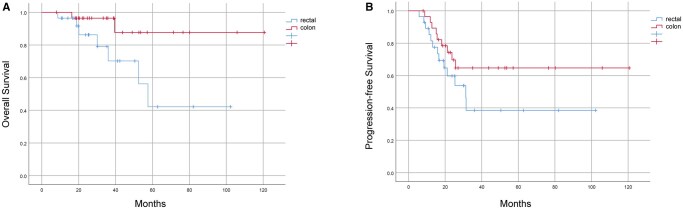
Kaplan–Meier survival curve for survival analysis in patients with mCRC and pCR of the primary tumor site based on the tumor location. (A) Overall survival and (B) progression-free survival. mCRC, metastatic colorectal cancer; pCR, pathological complete response.

The median follow-up was 27.1 (range, 7.9-120.6) months. A total of 22 (38.6%) patients had disease progression, while 9 (15.8%) patients died of cancer. Kaplan–Meier analysis revealed that the 2-year PFS and 3-year OS rates in the 57 cases were 62.2% and 84.5%, respectively. Compared to patients with rectal cancer (*n* = 28), patients with colon cancer (*n* = 29) had superior OS (*P* = .039) and a trend toward superior PFS (*P* = .149) (Figure 1). The 2-year PFS rate was 53.8% for patients with rectal cancer and 64.7% for those with colon cancer. (Tables 2 and 3).

**Table 2.** Univariate analyses of prognostic variables that correlated with OS.^a^

	Univariate analysis
Variable	HR (95% CI) *P* value
**Age**	0.991 (0.944-1.040) 0.711
**Sex**	
**Male**	Reference
**Female**	1.065 (0.284-3.990) 0.925
**Pretreatment CEA**	
**Normal**	Reference
**Elevated**	1.436 (0.384-5.366) 0.591
**Location**	
**Rectal**	Reference
**Colon**	0.220 (0.046-1.062) 0.059
**Surgical interval**	
**<180 days**	Reference
**≥180 days**	0.434 (0.088-2.144) 0.306
**Lymph node harvest**	0.972 (0.894-1.056) 0.501
**Targeted therapy**	
**No**	Reference
**Yes**	0.664 (0.162-2.724) 0.570
**Metastectomy**	
**No**	Reference
**Yes**	0.705 (0.187-2.662) 0.606
**Radiotherapy**	
**No**	Reference
**Yes**	1.952 (0.522-7.297) 0.320

a OS: overall survival.

**Table 3.** Univariate analysis of prognostic variables that correlated with PFS.^a^

	Univariate analysis
Variable	HR (95% CI) *P* value
**Age**	1.013 (0.986-1.039) 0.349
**Sex**	
**Male**	Reference
**Female**	0.673 (0.273-1.656) 0.388
**Pretreatment CEA**	
**Normal**	Reference
**Elevated**	0.898 (0.388-2.079) 0.801
**Location**	
**Rectal**	Reference
**Colon**	0.539 (0.230-1.264) 0.155
**Surgical interval**	
**<180 days**	Reference
**≥180 days**	1.098 (0.474-2.542) 0.828
**Lymph node harvest**	0.970 (0.918-1.025) 0.275
**KNB** ^b^ **status**	
**Wild type**	Reference
**Mutation**	1.996 (0.625-6.375) 0.244
**Unknown**	/
**Targeted therapy**	
**No**	Reference
**Yes**	1.152 (0.496-2.679) 0.742
**Metastectomy**	
**No**	Reference
**Yes**	0.569 (0.245-1.325) 0.191
**Radiotherapy**	
**No**	Reference
**Yes**	1.016 (0.396-2.604) 0.974

a PFS: progression-free survival.

b KNB: KRAS, NRAS, BRAF.

## Discussion

Neoadjuvant therapy is frequently used in the treatment of mCRC.^11^^,^^12^ However, pCR of the primary tumor site is rarely reported in patients with mCRC who received preoperative therapy and underwent surgical resection of the primary tumor. In this study, patients with mCRC who received preoperative therapy and surgery, with pathologically confirmed pCR of the primary tumor site, particularly those with colon cancer, have a favorable prognosis compared with the overall prognosis of mCRC reported in the literatures. The 2-year PFS and 3-year OS rates in this population were 62.2% and 84.5%, respectively. Therefore, this subset of patients should be identified in a timely manner, and the treatment goal should be more oriented toward curability.

A few studies have reported patients with colorectal cancer and dMMR/MSI-H who experienced a pCR following immunotherapy.^13–15^ Given the high sensitivity of this subgroup to immunotherapy, they were excluded from the present study to ensure our results would be specific to the more common pMMR/MSS mCRC population. To the best of our knowledge, this is the first study to report long-term survival in patients with mCRC with pCR of the primary lesion following preoperative chemotherapy or chemoradiotherapy. Prior research related to the ypT0N0M1 (primary pCR with synchronous metastases) subset of mCRC has been extremely limited, consisting primarily of isolated case reports or small case series.^16^^,^^17^ Thus, our study fills a critical gap in the literature by providing longitudinal survival data (median follow-up 27.1 months) for a cohort of 57 patients with pMMR/MSS mCRC and primary pCR after conventional neoadjuvant therapy.

The pCR of the primary CRC lesion often indicates favorable tumor biological behavior and better treatment response; therefore, a more proactive approach should be taken in the treatment of metastatic lesions. In this study, resection of metastatic lesions did not improve the prognosis of patients, which might be due to several possibilities. First, resection of only some lesions in cases of multifocal metastases sometimes fails to achieve a curative goal. Second, some patients who did not undergo resection of metastatic lesions might have achieved a curative goal through radiofrequency ablation. Third, some patients who did not undergo resection of metastatic lesions achieved complete clinical remission of metastatic lesions through drug therapy, thus exempting them from metastatic lesion surgery. Fourth, insufficient sample size may compromise the comparison of prognostic outcomes. Based on these possible reasons, no survival difference was found between the two groups when comparing those with resection of metastatic lesions vs. those without.

Systemic therapy is usually used for the neoadjuvant treatment of colon cancer, while systemic therapy in conjunction with local radiotherapy is often recommended for the treatment of rectal cancer. As a result, the pCR rate for rectal cancer is often significantly higher than that for colon cancer in locally advanced disease. pCR in colon cancer may thus more accurately reflect systemic therapy efficacy, whereas pCR in rectal cancer reflects combined systemic and local radiotherapy effects. This could explain why patients with colon cancer had better OS: systemic therapy that induces primary pCR may also more effectively control micrometastatic disease. However, this hypothesis requires validation in larger cohorts.

This study has several limitations. First, this study is a single-arm retrospective study without a control group and included only patients with pCR in the primary tumor. These patients had a relatively good response to treatment, which may lead to a certain selection bias. Second, with a sample size of only 57 cases, this study may lack the statistical power to detect differences in certain prognostic factors. Particularly, the lack of association between metastasectomy and survival may reflect limited statistical power, where small sample sizes increase the risk of Type II error. Third, the median follow-up period is relatively short at 27.1 months, which may underestimate non-cancer-related mortality, particularly given the upper follow-up range of 120.6 months. While no non-cancer deaths were observed to date, longer follow-up could reveal competing risks (eg, cardiovascular disease in older patients) that might alter OS estimates. Fourth, this study lacks systematic documentation for non–tumor-related deaths. All documented deaths (*n* = 9) were classified as related to cancer, with no available data on mortality from other causes (eg, treatment complications and comorbidities). This gap restricts our ability to evaluate competing risks, which are critical for interpreting OS in populations with metastatic cancer. Fifth, we did not collect data on all patients with mCRC who received systemic therapy followed by surgery (due to high time costs for subgroup data refinement) nor explore ypT0N0M1 within the broader mCRC context.

## Conclusion

In conclusion, patients with mCRC who received preoperative therapy and surgery, with pathologically confirmed pCR of the primary tumor site, particularly those with colon cancer have a favorable prognosis and should be identified promptly, and treatment goals should prioritize curability.

## Supplementary Material

oyag025_Supplementary_Data

## Data Availability

The data that support the findings of this study are available from the corresponding author upon reasonable request.
