# Supplementary figures and images for: Long-term outcomes following a pathological complete response at the primary tumor site after preoperative therapy in metastatic colorectal cancer
Source: Oncologist. 2026 Feb 3;31(4):oyag025. doi: 10.1093/oncolo/oyag025 (PMC12978306; doi:10.1093/oncolo/oyag025)

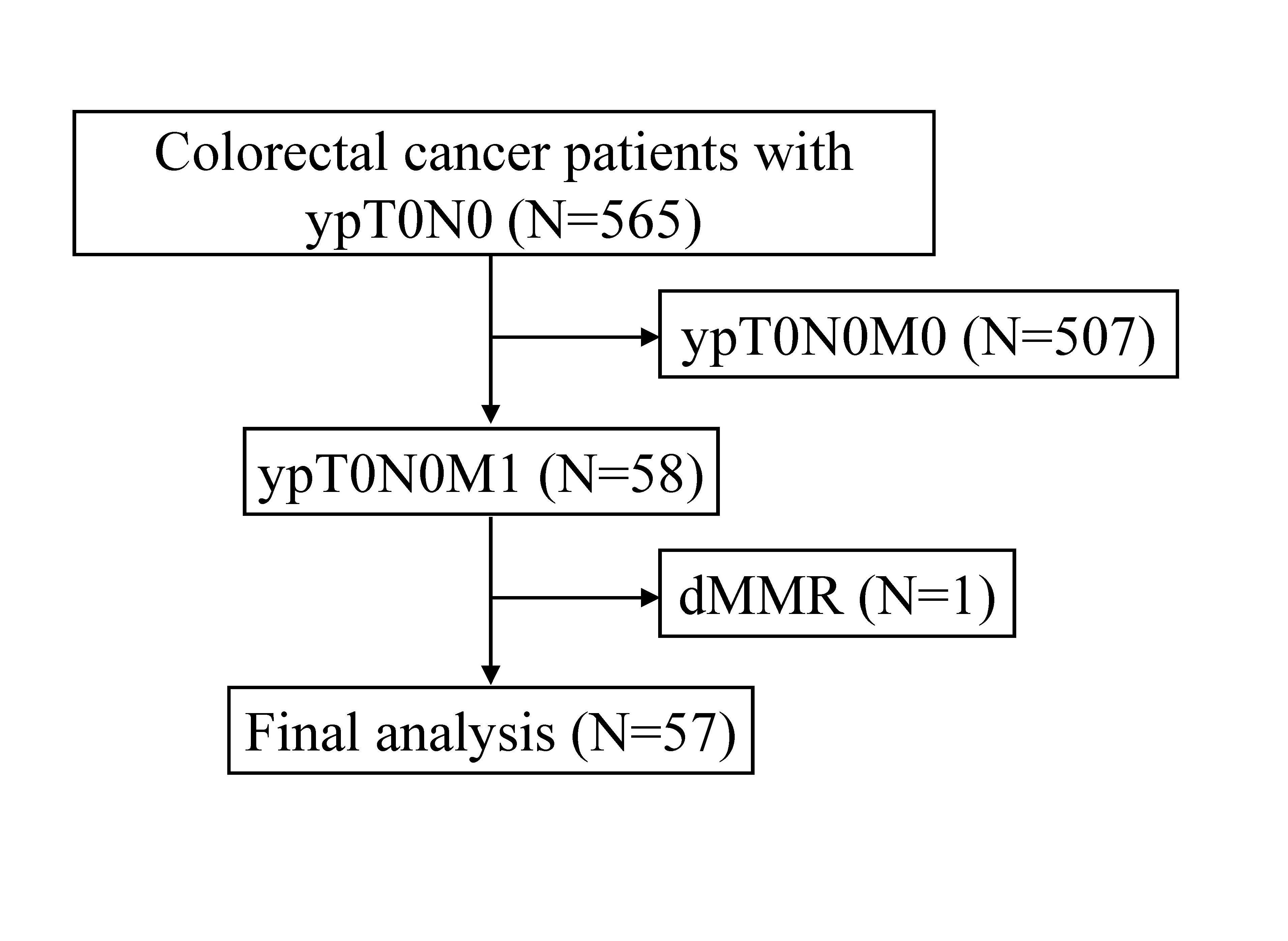

Supplement: oyag025_Supplementary_Data [file oyag025_supplementary_data.jpeg]
